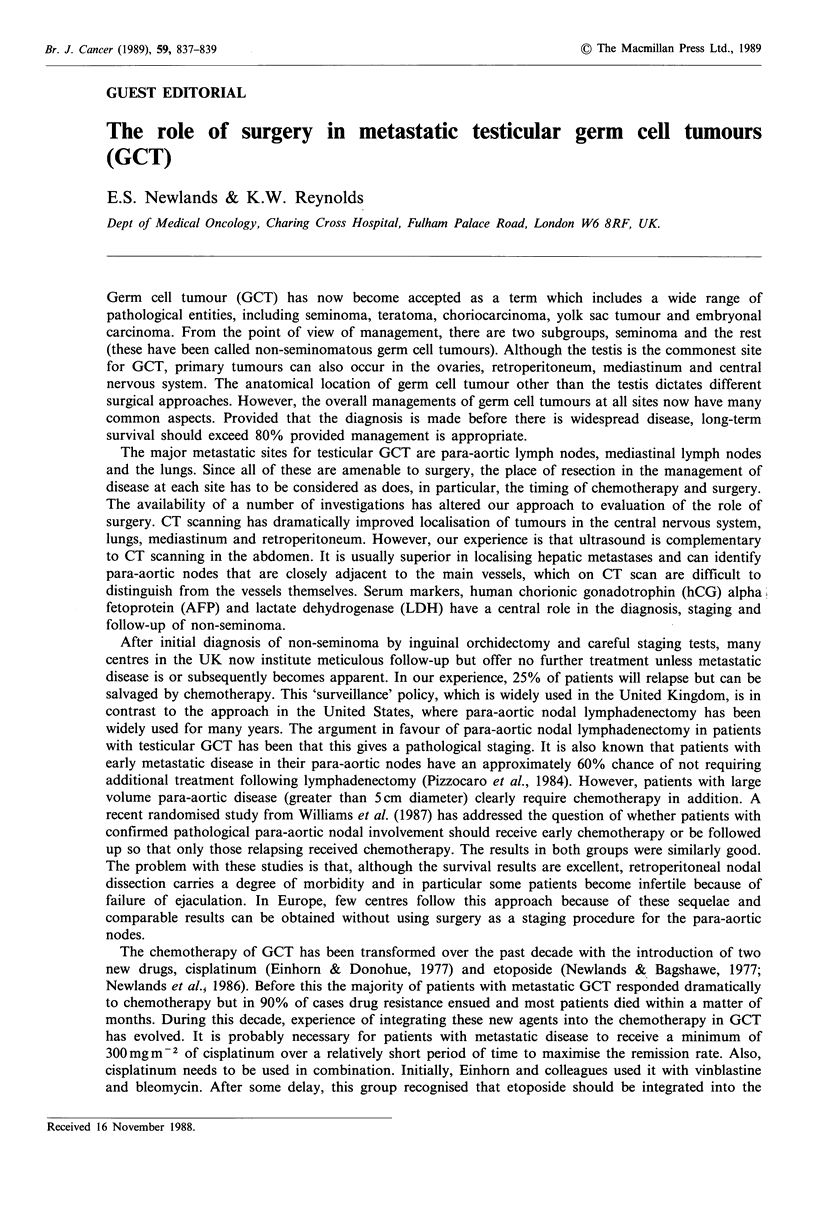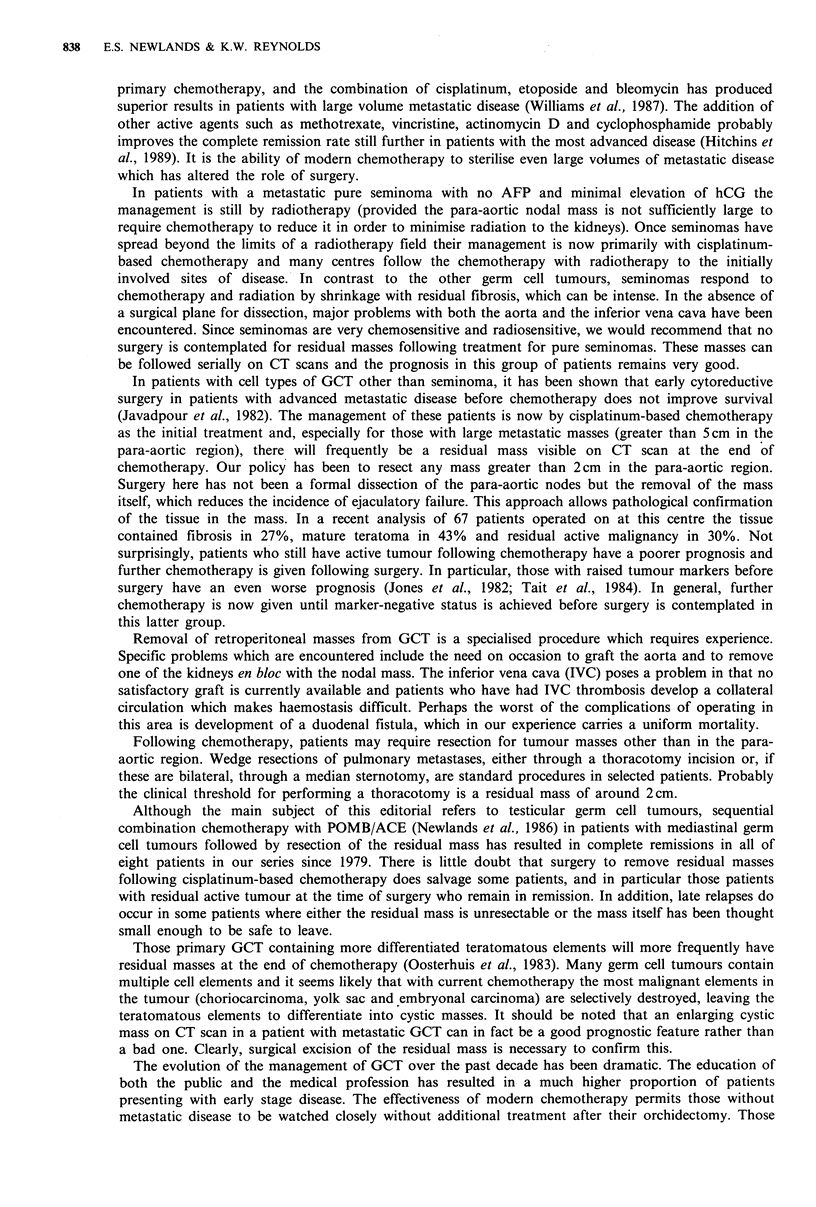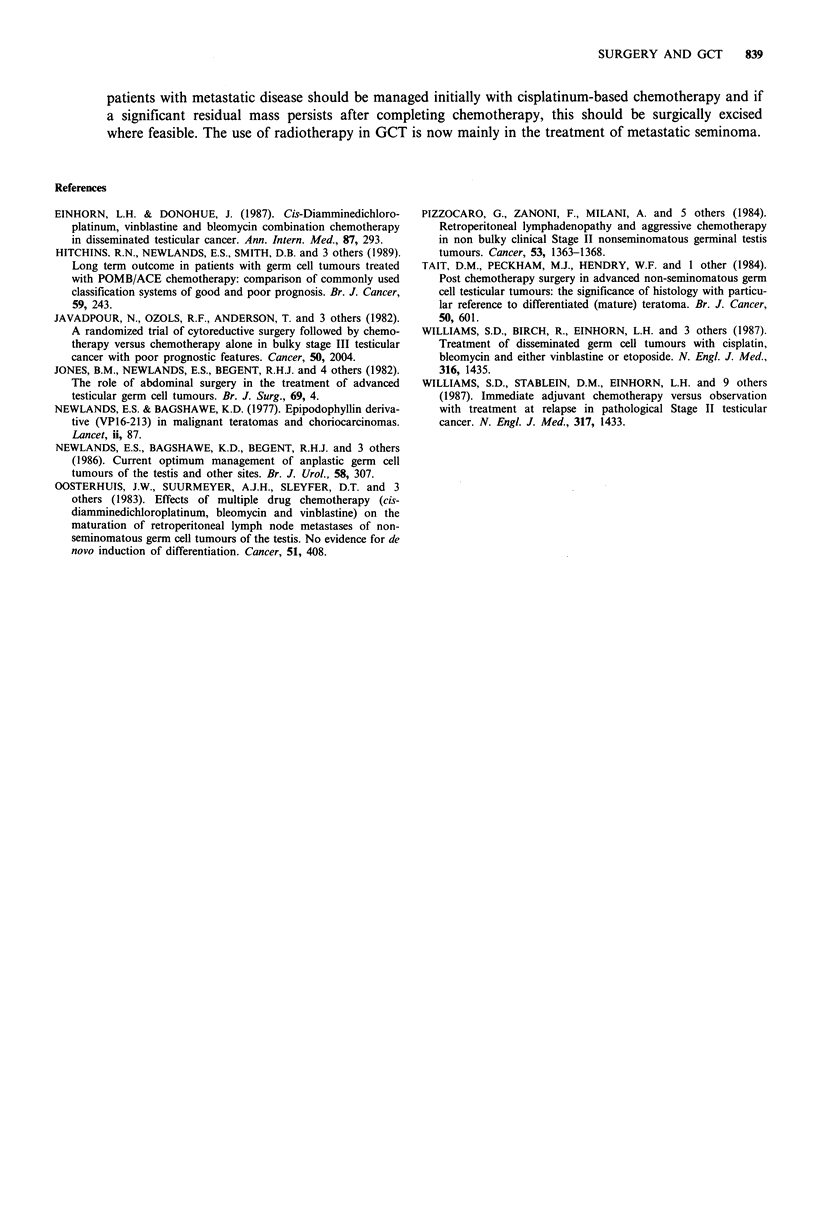# The role of surgery in metastatic testicular germ cell tumours (GCT).

**DOI:** 10.1038/bjc.1989.177

**Published:** 1989-06

**Authors:** E. S. Newlands, K. W. Reynolds

**Affiliations:** Dept of Medical Oncology, Charing Cross Hospital, London, UK.


Br. J. Cancer (1989), 59, 837–839 © The Macmillan Press Ltd., 1989

GUEST EDITORIAL

### The role of surgery in metastatic testicular germ cell tumours (GCT)

E.S. Newlands & K.W. Reynolds

*Dept of Medical Oncology, Charing Cross Hospital, Fulham Palace Road, London W6 8RF, UK.*

Germ cell tumour (GCT) has now become accepted as a term which includes a wide range of pathological entities, including seminoma, teratoma, choriocarcinoma, yolk sac tumour and embryonal carcinoma. From the point of view of management, there are two subgroups, seminoma and the rest (these have been called non-seminomatous germ cell tumours). Although the testis is the commonest site for GCT, primary tumours can also occur in the ovaries, retroperitoneum, mediastinum and central nervous system. The anatomical location of germ cell tumour other than the testis dictates different surgical approaches. However, the overall managements of germ cell tumours at all sites now have many common aspects. Provided that the diagnosis is made before there is widespread disease, long-term survival should exceed 80% provided management is appropriate.

The major metastatic sites for testicular GCT are para-aortic lymph nodes, mediastinal lymph nodes and the lungs. Since all of these are amenable to surgery, the place of resection in the management of disease at each site has to be considered as does, in particular, the timing of chemotherapy and surgery. The availability of a number of investigations has altered our approach to evaluation of the role of surgery. CT scanning has dramatically improved localisation of tumours in the central nervous system, lungs, mediastinum and retroperitoneum. However, our experience is that ultrasound is complementary to CT scanning in the abdomen. It is usually superior in localising hepatic metastases and can identify para-aortic nodes that are closely adjacent to the main vessels, which on CT scan are difficult to distinguish from the vessels themselves. Serum markers, human chorionic gonadotrophin (hCG) alpha fetoprotein (AFP) and lactate dehydrogenase (LDH) have a central role in the diagnosis, staging and follow-up of non-seminoma.

After initial diagnosis of non-seminoma by inguinal orchidectomy and careful staging tests, many centres in the UK now institute meticulous follow-up but offer no further treatment unless metastatic disease is or subsequently becomes apparent. In our experience, 25% of patients will relapse but can be salvaged by chemotherapy. This 'surveillance' policy, which is widely used in the United Kingdom, is in contrast to the approach in the United States, where para-aortic nodal lymphadenectomy has been widely used for many years. The argument in favour of para-aortic nodal lymphadenectomy in patients with testicular GCT has been that this gives a pathological staging. It is also known that patients with early metastatic disease in their para-aortic nodes have an approximately 60% chance of not requiring additional treatment following lymphadenectomy (Pizzocaro *et al.*, 1984). However, patients with large volume para-aortic disease (greater than 5 cm diameter) clearly require chemotherapy in addition. A recent randomised study from Williams *et al.* (1987) has addressed the question of whether patients with confirmed pathological para-aortic nodal involvement should receive early chemotherapy or be followed up so that only those relapsing received chemotherapy. The results in both groups were similarly good. The problem with these studies is that, although the survival results are excellent, retroperitoneal nodal dissection carries a degree of morbidity and in particular some patients become infertile because of failure of ejaculation. In Europe, few centres follow this approach because of these sequelae and comparable results can be obtained without using surgery as a staging procedure for the para-aortic nodes.

The chemotherapy of GCT has been transformed over the past decade with the introduction of two new drugs, cisplatinum (Einhorn & Donohue, 1977) and etoposide (Newlands & Bagshawe, 1977; Newlands *et al.*, 1986). Before this the majority of patients with metastatic GCT responded dramatically to chemotherapy but in 90% of cases drug resistance ensued and most patients died within a matter of months. During this decade, experience of integrating these new agents into the chemotherapy in GCT has evolved. It is probably necessary for patients with metastatic disease to receive a minimum of 300 mg m$^{-2}$ of cisplatinum over a relatively short period of time to maximise the remission rate. Also, cisplatinum needs to be used in combination. Initially, Einhorn and colleagues used it with vinblastine and bleomycin. After some delay, this group recognised that etoposide should be integrated into the

Received 16 November 1988.

primary chemotherapy, and the combination of cisplatinum, etoposide and bleomycin has produced superior results in patients with large volume metastatic disease (Williams *et al.*, 1987). The addition of other active agents such as methotrexate, vincristine, actinomycin D and cyclophosphamide probably improves the complete remission rate still further in patients with the most advanced disease (Hitchins *et al.*, 1989). It is the ability of modern chemotherapy to sterilise even large volumes of metastatic disease which has altered the role of surgery.

In patients with a metastatic pure seminoma with no AFP and minimal elevation of hCG the management is still by radiotherapy (provided the para-aortic nodal mass is not sufficiently large to require chemotherapy to reduce it in order to minimise radiation to the kidneys). Once seminomas have spread beyond the limits of a radiotherapy field their management is now primarily with cisplatinum-based chemotherapy and many centres follow the chemotherapy with radiotherapy to the initially involved sites of disease. In contrast to the other germ cell tumours, seminomas respond to chemotherapy and radiation by shrinkage with residual fibrosis, which can be intense. In the absence of a surgical plane for dissection, major problems with both the aorta and the inferior vena cava have been encountered. Since seminomas are very chemosensitive and radiosensitive, we would recommend that no surgery is contemplated for residual masses following treatment for pure seminomas. These masses can be followed serially on CT scans and the prognosis in this group of patients remains very good.

In patients with cell types of GCT other than seminoma, it has been shown that early cytoreductive surgery in patients with advanced metastatic disease before chemotherapy does not improve survival (Javadpour *et al.*, 1982). The management of these patients is now by cisplatinum-based chemotherapy as the initial treatment and, especially for those with large metastatic masses (greater than 5 cm in the para-aortic region), there will frequently be a residual mass visible on CT scan at the end of chemotherapy. Our policy has been to resect any mass greater than 2 cm in the para-aortic region. Surgery here has not been a formal dissection of the para-aortic nodes but the removal of the mass itself, which reduces the incidence of ejaculatory failure. This approach allows pathological confirmation of the tissue in the mass. In a recent analysis of 67 patients operated on at this centre the tissue contained fibrosis in 27%, mature teratoma in 43% and residual active malignancy in 30%. Not surprisingly, patients who still have active tumour following chemotherapy have a poorer prognosis and further chemotherapy is given following surgery. In particular, those with raised tumour markers before surgery have an even worse prognosis (Jones *et al.*, 1982; Tait *et al.*, 1984). In general, further chemotherapy is now given until marker-negative status is achieved before surgery is contemplated in this latter group.

Removal of retroperitoneal masses from GCT is a specialised procedure which requires experience. Specific problems which are encountered include the need on occasion to graft the aorta and to remove one of the kidneys *en bloc* with the nodal mass. The inferior vena cava (IVC) poses a problem in that no satisfactory graft is currently available and patients who have had IVC thrombosis develop a collateral circulation which makes haemostasis difficult. Perhaps the worst of the complications of operating in this area is development of a duodenal fistula, which in our experience carries a uniform mortality.

Following chemotherapy, patients may require resection for tumour masses other than in the para-aortic region. Wedge resections of pulmonary metastases, either through a thoracotomy incision or, if these are bilateral, through a median sternotomy, are standard procedures in selected patients. Probably the clinical threshold for performing a thoracotomy is a residual mass of around 2 cm.

Although the main subject of this editorial refers to testicular germ cell tumours, sequential combination chemotherapy with POMB/ACE (Newlands *et al.*, 1986) in patients with mediastinal germ cell tumours followed by resection of the residual mass has resulted in complete remissions in all of eight patients in our series since 1979. There is little doubt that surgery to remove residual masses following cisplatinum-based chemotherapy does salvage some patients, and in particular those patients with residual active tumour at the time of surgery who remain in remission. In addition, late relapses do occur in some patients where either the residual mass is unresectable or the mass itself has been thought small enough to be safe to leave.

Those primary GCT containing more differentiated teratomatous elements will more frequently have residual masses at the end of chemotherapy (Oosterhuis *et al.*, 1983). Many germ cell tumours contain multiple cell elements and it seems likely that with current chemotherapy the most malignant elements in the tumour (choriocarcinoma, yolk sac and embryonal carcinoma) are selectively destroyed, leaving the teratomatous elements to differentiate into cystic masses. It should be noted that an enlarging cystic mass on CT scan in a patient with metastatic GCT can in fact be a good prognostic feature rather than a bad one. Clearly, surgical excision of the residual mass is necessary to confirm this.

The evolution of the management of GCT over the past decade has been dramatic. The education of both the public and the medical profession has resulted in a much higher proportion of patients presenting with early stage disease. The effectiveness of modern chemotherapy permits those without metastatic disease to be watched closely without additional treatment after their orchidectomy. Those patients with metastatic disease should be managed initially with cisplatinum-based chemotherapy and if a significant residual mass persists after completing chemotherapy, this should be surgically excised where feasible. The use of radiotherapy in GCT is now mainly in the treatment of metastatic seminoma.

### References

EINHORN, L.H. & DONOHUE, J. (1987). *Cis*-Diamminedichloroplatinum, vinblastine and bleomycin combination chemotherapy in disseminated testicular cancer. *Ann. Intern. Med.*, **87,** 293.

HITCHINS. R.N., NEWLANDS, E.S., SMITH, D.B. and 3 others (1989). Long term outcome in patients with germ cell tumours treated with POMB/ACE chemotherapy: comparison of commonly used classification systems of good and poor prognosis. *Br. J. Cancer*, **59,** 243.

JAVADPOUR, N., OZOLS, R.F., ANDERSON, T. and 3 others (1982). A randomized trial of cytoreductive surgery followed by chemotherapy versus chemotherapy alone in bulky stage III testicular cancer with poor prognostic features. *Cancer*, **50,** 2004.

JONES, B.M., NEWLANDS, E.S., BEGENT, R.H.J. and 4 others (1982). The role of abdominal surgery in the treatment of advanced testicular germ cell tumours. *Br. J. Surg.*, **69,** 4.

NEWLANDS, E.S. & BAGSHAWE, K.D. (1977). Epipodophyllin derivative (VP16-213) in malignant teratomas and choriocarcinomas. *Lancet*, **ii,** 87.

NEWLANDS, E.S., BAGSHAWE, K.D., BEGENT, R.H.J. and 3 others (1986). Current optimum management of anplastic germ cell tumours of the testis and other sites. *Br. J. Urol.*, **58,** 307.

OOSTERHUIS, J.W., SUURMEYER, A.J.H., SLEYFER, D.T. and 3 others (1983). Effects of multiple drug chemotherapy (*cis*-diamminedichloroplatinum, bleomycin and vinblastine) on the maturation of retroperitoneal lymph node metastases of non-seminomatous germ cell tumours of the testis. No evidence for *de novo* induction of differentiation. *Cancer*, **51,** 408.

PIZZOCARO, G., ZANONI, F., MILANI, A. and 5 others (1984). Retroperitoneal lymphadenopathy and aggressive chemotherapy in non bulky clinical Stage II nonseminomatous germinal testis tumours. *Cancer*, **53,** 1363–1368.

TAIT, D.M., PECKHAM, M.J., HENDRY, W.F. and 1 other (1984). Post chemotherapy surgery in advanced non-seminomatous germ cell testicular tumours: the significance of histology with particular reference to differentiated (mature) teratoma. *Br. J. Cancer*, **50,** 601.

WILLIAMS, S.D., BIRCH, R., EINHORN, L.H. and 3 others (1987). Treatment of disseminated germ cell tumours with cisplatin, bleomycin and either vinblastine or etoposide. *N. Engl. J. Med.*, **316,** 1435.

WILLIAMS, S.D., STABLEIN, D.M., EINHORN, L.H. and 9 others (1987). Immediate adjuvant chemotherapy versus observation with treatment at relapse in pathological Stage II testicular cancer. *N. Engl. J. Med.*, **317,** 1433.